# Immune-Related lncRNAs to Construct Novel Signatures and Predict the Prognosis of Rectal Cancer

**DOI:** 10.3389/fonc.2021.661846

**Published:** 2021-08-16

**Authors:** Xiao-Liang Xing, Chaoqun Xing, Zhi Huang, Zhi-Yong Yao, Yuan-Wu Liu

**Affiliations:** ^1^School of Public Health and Laboratory Medicine, Hunan University of Medicine, Huaihua, China; ^2^Department of Pediatrics, Xiangya Hospital, Central South University, Changsha, China; ^3^Beijing Advanced Innovation Center for Food Nutrition and Human Health, China Agricultural University, Beijing, China

**Keywords:** READ, immune, LncRNAs, overall survival, risk model

## Abstract

Colorectal cancer (CRC) is one of the most common cancers. Almost 1/3 of CRC are rectal cancer, and 95% of rectal cancers are rectal adenocarcinoma (READ). Increasing evidences indicated that long noncoding RNAs (lncRNAs) have important role in the genesis and development of cancers. The purpose of our present study was to identify the differential expression lncRNAs which potentially related with immune cells infiltration and establish a risk assessment model to predict the clinical outcome for READ patients. We obtained three immune-related differential expression lncRNAs (IR-DELs) (C17orf77, GATA2-AS1, and TPT1-AS1) by differential expression analysis following correlation analysis and Cox regression analysis. A risk assessment model were constructed by integrating these analysis results. We then plotted the 1-, 3-, and 5-year ROC curves depending on our risk assessment model, which suggested that all AUC values were over 0.7. In addition, we found that the risk assessment model was correlated with several immune cells and factors. This study suggested that those three signatures (C17orf77, GATA2-AS1, and TPT1-AS1) screened by pairing IR-DELs could be prognosis markers for READ patients and might benefit them from antitumor immunotherapy.

## Introduction

Colorectal cancer (CRC) is one of the most prevalent cancer which accounts for approximately 10% of cancer cases and deaths annually. There are almost 1.8 million new cases of CRC and 881,000 deaths occurred in the world globally (1). Rectal cancer as a subtype of CRC which accounts for one-third of newly diagnosed CRC cases every year (2); 95% of rectal cancer is the rectal adenocarcinoma (READ). The treatment guidelines state that preoperative neoadjuvant chemotherapy could improve the radical resection rate and reduce local recurrence. Thus, even though surgery is the primary treatment for READ, preoperative neoadjuvant chemoradiotherapy is still the key to the treatment of READ (3, 4). However, previous studies demonstrated that almost 30% of patients treated with a curative treatment will develop distant metastases finally (5–8). It is very important to find suitable biomarkers for the prognosis of READ patients.

Recently, increasing evidences indicated that immune cells and immune factors participated in antitumor effects, including tumor initiation and progression (9, 10). The immunotherapy has promising advantages for cancers in the treatment efficiency and long-term survival of patients (11). CRC is a type of tumor which is accompanied by high density of tumor-infiltrating lymphocytes. The CRC patients, for example, with higher CD8^+^ T lymphocytes always displayed a better prognosis (12–14). Actually, previous studies demonstrated that signatures focusing on the tumor immune infiltrations have shown a promising predictive and prognostic value in diagnosis, evaluation, and treatment of cancer (15).

Long noncoding RNAs (lncRNAs) are kind of RNA which not coding for any protein or peptides and account for approximately 80% of the human transcriptome. Most of lncRNAs are more than 200 nucleotides and regulate 70% of the gene expression. In recent years, increasing studies have shown that lncRNAs are deeply involved in both normal cell development and cancer genesis (16, 17). In the present study, we aimed to investigate the relationships of lncRNAs with gene expression profile and the tumor-immune microenvironment in READ, and construct a risk assessment model for prognosis prediction in READ patients and benefit them from antitumor immunotherapy.

## Material and Method

### Data Source and Differentially Expression Analysis

The data used in this study was obtained from openly database TCGA (The Cancer Genome Atlas), which included 10 normal and 166 READ samples. The mRNA expression profile data were analyzed by DESeq2 package in R (3.6.1) software as cutoff with padj <0.05, |logFC| ≥0.5, and basemean >50. Gene transfer format (GTF) files were downloaded from Ensembl (http://asia.ensembl.org) for gene annotation. A list of recognized immune-related genes (IR-Genes) was downloaded from the ImmPort database (http://www.immport.org) and was used to screen IR-lncRNAs by a coexpression strategy. The extent of immune cell infiltration was obtained from Tumor Immune Estimation Resource (TIMER) (https://cistrome.shinyapps.io/timer/). Spearman’s correlation analysis was performed between IR-DEGs and their corresponding lncRNAs as cutoff with *r* ≥0.3 and *p*-value <0.05 to screen the immune-related differentially expressed lncRNAs (IR-DELs).

### Survival Analysis

According to the median expression value or median risk score, patients were divided into high-expression/risk group and low-expression/risk group. Survival, Survminer, and RegParallel package in R were used to carry out univariate and multivariate Cox regression analysis.

### Construction of Specific Risk Assessment Model

The risk model was established based on the expression data and multiplied Cox regression coefficients. The formula was set as followed, risk score = expression level of C17orf77 * (−1.5905) + expression levels of GATA2-AS1 * (−1.3003) + expression level of TPT1-AS1 * (−1.4072) (18, 19). To further investigate the relevance of the risk model with the clinical pathological features of READ, we analyzed the relationship between the risk value and vital status, gender, pathologic NMT, and pathologic stage. We then constructed time-dependent receiver operating characteristic (ROC) curves within 1-year, 3-year, 5-year, and clinical features and estimated its utility as a prognostic model for predicting the survival status.

### Statistical Analysis

A repeated-measure ANOVA followed by Bonferroni *post hoc* tests or unpaired two-tail Student’s *t*-test was used as indicated. All statistical analyses were performed using the Graphpad Prism 6.0.1.

## Results

### Identification of Immune-Related Differentially Expressed lncRNAs

**Figure 1:**
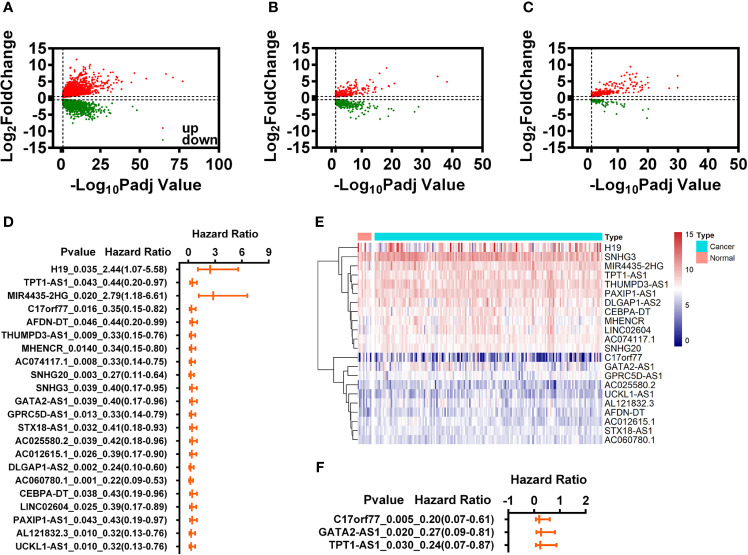
Identification of immune-related differential expression lncRNAs (IR-DELs). **(A)** Volcano plot of DEGs for READ. **(B)** Volcano plot of IR-DEGs for READ. **(C)** Volcano plot of DELs for READ. **(D)** The univariate Cox regression analysis for those 22 IR-DELs. **(E)** The heatmap of 22 IR-DELs verified by univariate Cox regression analysis. **(F)** The multivariate Cox regression analysis for those three IR-DELs.

We downloaded the expression data of 176 samples (10 control *vs.* 166 READ) from TCGA database. Through differential expression analysis by DEseq2, we screened 7,283 differentially expression genes (DEGs) (4,120 DEGs were upregulated and 3,163 DEGs were downregulated) (**Figure 1A**). Of which, we obtained 514 immune-related DEGs (IR-DEGs) by cross-analysis with the recognized immune-related genes (**Figure 1B**) and 420 differential expression lncRNAs (DELs) by cross-analysis with GTF annotation data (**Figure 1C**). We then introduced the Spearman’s correlation analysis for those 514 IR-DEGs and 420 DELs and identified that there were 9,973 IR-DEGs-IR-DELs pairs involved 508 IR-DEGs and 409 IR-DELs.

The purpose of this study was to identify the IR-DELs which could use to construct a risk assessment model to predict the outcome of READ. Therefore, we performed univariate Cox regression analysis for those 409 IR-DELs and found 22 IR-DEL expression were associated with the overall survival (OS) of READ (**Figure 1D**). The expressions of those DELs are shown in **Figure 1E**. Subsequently, we also performed multivariate Cox regression analysis for those 22 IR DELs and found three IR-DELs (C17orf77, GATA2-AS1, and TPT1-AS1) were still associated with the OS of READ (**Figure 1F**).

### Establishment of Specific Risk Assessment Model

**Figure 2:**
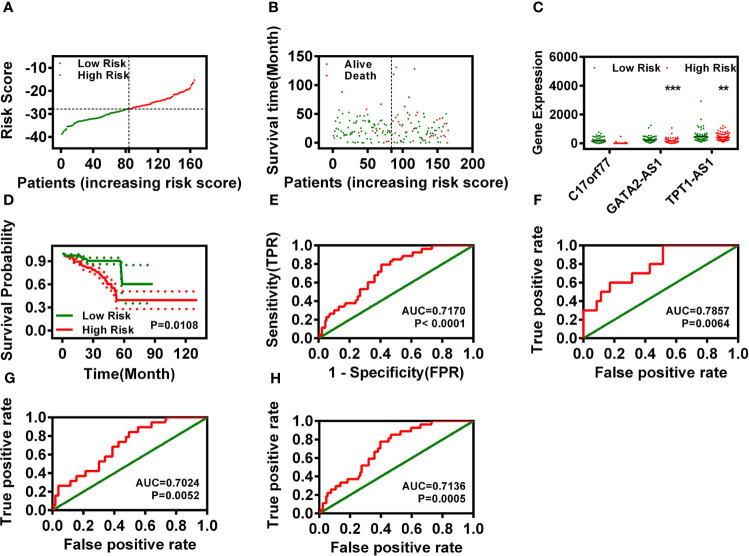
Risk assessment model construction based on three signature DELs. **(A, B)** Risk scores **(A)** and survival outcome **(B)** of each case for READ. **(C)** The expression of C17orf77, GATA2-AS1, and TPT1-AS1 between low- and high-risk groups. **(D)** Overall survival analysis of the risk model. **(E)** The ROC curve of the risk model. **(F–H)** The 1-year **(F)**, 3-year **(G)**, and 5-year **(H)** ROC of the prognosis model suggested that all AUC values were over 0.70.

The expressions of those three DELs (C17orf77, GATA2-AS1, and TPT1-AS1) were increased significantly in the cancer group (**Figure 1E**). We used those three DELs to construct a specific risk assessment model. The risk scores and survival outcome of each READ cases are displayed in **Figures 2A, B**. The expression of C17orf77 and GATA2-AS1 were decreased significantly while the expression of TPT1-AS1 was comparable between the low-r and high-risk groups (**Figure 2C**).

After regrouping the READ patients by risk value, we analyzed the relationship of the risk model with the OS of READ and found that low-risk READ patients exhibited a better OS (**Figure 2D**). Meanwhile, we also calculated the areas under curve (AUCs) for each receiver operating characteristic (ROC) curve of those three IR-DELs and drew the curved line (**Figure 2E**). To validate the optimality, we also plotted the 1-, 3-, and 5-year ROC curves, which suggested that all AUC values were over 0.7 (**Figures 2F–H**).

### Clinical Evaluation by Risk Assessment Model

**Figure 3:**
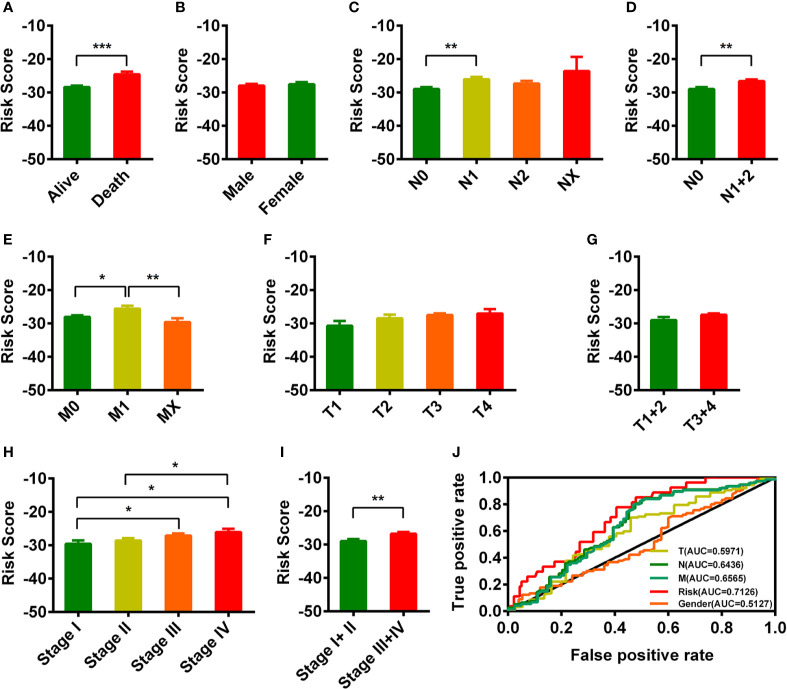
Clinical evaluations with the risk model. **(A–I)** The histogram showed that vital status **(A)**, pathologic N **(C**, **D)**, pathologic M **(E)**, and pathologic stage **(H, I)** were significantly associated with the risk score. The risk score in different gender groups **(B)** and different pathologic T groups **(F, G)** were comparable. The lower-risk score represented lower risk in prognosis. **(J)** The AUC value of TNM staging, gender, and risk model. *p < 0.05; **p < 0.01; ***p < 0 .001.

The TNM staging system was widely used for cancer stage classification and determining the appropriate treatment strategy. Therefore, we analyzed the relationship of the risk score with the clinical characteristics. As shown in **Figure 3**, we found that the risk score in the death READ patients was significantly higher than that in the alive READ patients (**Figure 3A**). There was a comparable risk value between male and female (**Figure 3B**). The risk score showed several differences among pathologic N, pathologic M, and pathologic stage system except pathologic T system (**Figures 3C–I**). We also calculated the AUC for each ROC curve of those clinical characters drew the curved line; the result is shown in **Figure 3J**. The AUC value of the risk model in predicting the survival status was the highest (0.7126).

### Estimation of Tumor-Infiltration With the Risk Assessment Model

**Figure 4:**
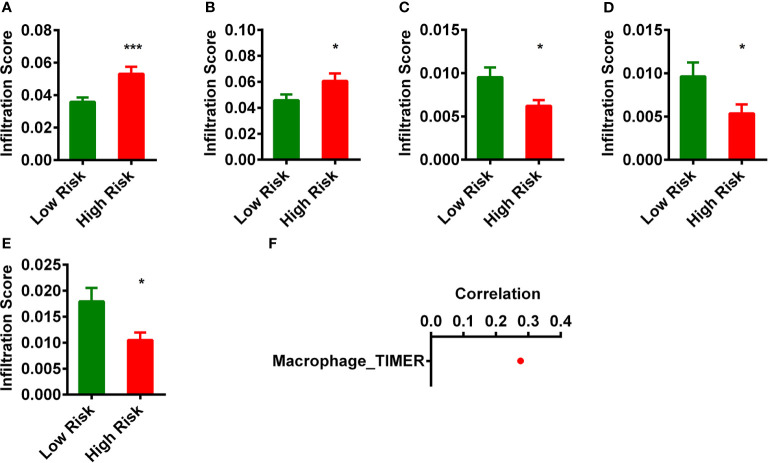
Estimation of tumor-infiltrating immune cells with risk assessment model. **(A–E)** The infiltrating score of five immune cells and immune factors **(A)**, Macrophage_TIMER; **(B)**, Macrophage M0_CIBERSORT-ABS. **(C)**, B-cell plasma_XCELL. **(D)**, Class-switched memory B cell_XCELL. **(E)**, Plasmacytoid dendritic cell_XCELL) between low- and high-risk groups were difference significantly. **(F)** Patients in the high-risk group were more positively associated with Macrophage_TIMER. *p < 0.05; ***p < 0.001.

Firstly, we analyzed the differences of immune infiltration between normal and READ patients and found that there are 50 immune cells and factors that were different between control and cancer. Because lncRNAs and immune-related genes were initially connected, we consequently investigated the relationship of the risk model with the tumor immune microenvironment (**Supplementary Table 1**). We then investigated whether those 64 immune cells and factors were different between low-risk group and high-risk group. After this analysis, we found five immune cells and immune factors were different (**Figures 4A–E**). A detailed Spearman’s correlation analysis was conducted; the result is shown in **Figure 4F**. READ patients in the high-risk group were more positively associated with Macrophage_TIMER.

## Discussions

CRC is one of the most common cancers which comprised colon cancer and rectal cancer. While colon cancer and rectal cancer are very similar in several ways, their treatments are quite different. Due to the improvement of the treatment over the last few decades, the OS rate of READ has greatly improved. However, the additional long-term survival for READ patients is hard to achieve even under extensive treatment compared with other cancers. Previous studies indicated that almost 30% of patients treated with a curative treatment will eventually develop distant metastases (5–8). Therefore, the early diagnosis and prediction of prognosis in patients with READ are important for their long-term survival.

Increasing evidence indicated that lncRNAs deeply participated in the tumor genesis, development, and metastasis (20–22). In another aspect, lncRNAs were screened to be the prognosis biomarkers for cancers, including READ. Chao et al. (2019) found lncRNA D16366 could be a potential biomarker for diagnosis and prognosis of hepatocellular carcinoma (23). Zhao et al. (23) found that five lncRNAs (AC079789.1, AC106900.2, AL121987.1, AP004609.1, and LINC02163) were independently associated with the prognosis of patients with rectal cancer through using univariate and multivariate Cox regression analyses (24). Those five lncRNAs could be the potential biomarkers for READ (24). Qi et al. (25) found that lncRNA KCNQ1OT1 and SNHG1 were unveiled as common diagnostic biomarkers for the initiation and metastasis of colon and rectal cancers (25).

Increasing evidences indicated that immune cells and factors play an important role in antitumor immunity and antitumor initiation and progression (9, 10). Immune checkpoint blockade therapy has become an important cancer treatment option and immunotherapy has promising advantages in terms of efficiency and long-term survival (11). Antibody drugs, such as anti-PD-1 and anti-PD-L1, demonstrate obvious advantages such as broad applicability across cancer types and durable clinical response when treatment is effective (26). In the present study, we aimed to investigate the relationships of IR-DELs with immune cells and immune factors of READ and construct a risk assessment model to predict the prognosis for patients with READ and distinguishing those who could benefit from antitumor immunotherapy. First, we found that 23 IR-DELs were associated with the OS of READ by differential expression analysis, correlation analysis, and univariate Cox regression analysis. Three IR-DELs (C17orf77, GATA2-AS1, and TPT1-AS1) were independently associated with the OS of READ. Previous studies indicated that OS-related genes could be used to construct the risk assessment model. According to previous studies, we also constructed a risk assessment model by using those three IR-DELs (C17orf77, GATA2-AS1, and TPT1-AS1) (18, 19). The correlation analysis results showed that risk value was correlated with pathologic N, pathologic M, pathologic stage, several immune cells, and immune factors. The AUC value was over 0.7 which proved that the model had certain accuracy in predicting the prognosis. However, whether this model could be used for clinical prognosis, diagnosis still needs further validation studies.

Chromosome 17 Open Reading Frame 77 (*C17orf77*) is a gene in cancer that has not been well studied yet. In 2019, Shaikh et al. found that C17orf77 may hold a putative role in both pathogenesis of smoking and nonsmoking-related head and neck squamous cell carcinoma tumors and could be considered a potential biomarker for separating these tumors (27). GATA-binding protein 2 (GATA2) is a member of the GATA family of zinc-finger transcription factors. GATA2 exists as an acetylated protein in immature precursor cells. GATA2-AS1 is the antisense RNA of GATA2. In 2019, Zhang et al. found that GATA2-AS1 could repress nonsmall-cell lung cancer cell proliferation *via* regulating GATA2. These results indicated that GATA2-AS1 could be a potential target for lung cancer drugs (28). In 2020, Li et al. found that GATA2-AS1 was a tumor-associated lnRNAs in COAD by bioinformatics analysis (29). TPT1-AS1 is the antisense RNA of tumor protein, translationally controlled 1 (TPT1). Previous studies indicated that TPT1-AS1 could regulate the genesis and development of several cancers by different ways. Huang et al. and Gao et al. found that TPT1-AS1 could promote cell growth in cervical cancer and glioblastoma *via* acting as a sponge for miR-324-5p and miR-23a-5p, respectively (30, 31). Tumors grow and evolve through constant crosstalk with the surrounding microenvironment, and emerging evidences indicate that angiogenesis and immunosuppression frequently occur simultaneously in response to this crosstalk. Although VEGFR2 was downregulated in the prostate cancer, VEGFR2 was upregulated in the high-risk prostate cancer and predicted clinical progression. Krebs et al. found miR-221-3p upregulation as an escape mechanism from VEGFR2 inhibition in prostate cells (32). Zhang et al. (33) and Zhang et al. (34) found that TPT1-AS1 could promote angiogenesis, progression, and metastasis of CRC through TPT1-AS1/NF90/VEGFA signaling pathway and upregulating the TPT1-mediated FAK and JAK-STAT3 signaling pathways, respectively (33, 34). Our present study indicated that C17orf77, GATA2-AS1, and TPT1-AS1 could be the prognosis biomarkers for READ which reinforced the role of those lncRNAs in predicting the outcome of cancers as biomarkers.

In conclusion, we identified three IR-DELs correlated with OS and constructed a specific risk assessment model for prognostic prediction of READ. Those three IR-DEL classifiers also demonstrated considerable predictive accuracy for predicting the OS. In addition, the risk model was correlated with immunotherapy-related biomarkers, suggesting its application value for predicting the efficiency of treatment. However, we also recognized some shortcomings and limitations in this study, especially for the lack of clinical validation. Therefore, we will collect clinical samples and carry out the verification in the future work.

## Data Availability Statement

Publicly available datasets were analyzed in this study. This data can be found here: https://portal.gdc.cancer.gov/.

## Author Contributions

Z-YY and Y-WL conceived and designed the experiments. X-LX performed the analysis. CX and ZH helped to analyze the data. Y-WL and X-LX wrote the paper. All authors contributed to the article and approved the submitted version.

## Funding

This project is financially supported by the Doctor Foundation of Hunan University of Medicine (2020122004), China Hunan Provincial Science and Technology Department (2020SK4004), and China Hunan Provincial Education Department (20C1328).

## Conflict of Interest

The authors declare that the research was conducted in the absence of any commercial or financial relationships that could be construed as a potential conflict of interest.

## Publisher’s Note

All claims expressed in this article are solely those of the authors and do not necessarily represent those of their affiliated organizations, or those of the publisher, the editors and the reviewers. Any product that may be evaluated in this article, or claim that may be made by its manufacturer, is not guaranteed or endorsed by the publisher.
