# Supplementary material for: Immune-Related lncRNAs to Construct Novel Signatures and Predict the Prognosis of Rectal Cancer
Source: Front Oncol. 2021 Aug 16;11:661846. doi: 10.3389/fonc.2021.661846 (PMC8415501; doi:10.3389/fonc.2021.661846)
Supplement: Supplementary file 1 [file DataSheet_1.pdf]

Immune-Related lncRNAs to Construct Novel Signature and Predict the Prognosis of Rectal Cancer

Xiao-Liang Xing[1,2], Chaoqun Xing[1,2], Zhi Huang[1,2], Zhi-Yong Yao[1,2*], Yuan-Wu Liu[3*]

[1]School of public health and laboratory medicine, Hunan University of Medicine, Huaihua 418000, Hunan, P. R. China.

[2]Xiangya Hospital, Central South University, Changsha, 410078, Hunan, P. R. China.

[3]Beijing Advanced Innovation Center for Food Nutrition and Human Health, China Agricultural University, 100193, Beijing, China.

*Correspondence: Zhi-Yong Yao, 179129382@qq.com. Yuan-Wu Liu, yuanwu_liu@126.com.

Supplementary table 1 The significantly difference immune cells and immune factors between control and cancer

Type	Immune cells and factors	Control (n=10)		READ (n=166)	
		Mean	SD	Mean	SD
CIBERSORT	Mast cell activated	0.07	0.05	0.01	0.02
	B cell plasma	0.14	0.06	0.05	0.05
	Macrophage M0	0.00	0.00	0.14	0.10
	B cell naive	0.06	0.05	0.02	0.03
	Macrophage M1	0.01	0.01	0.05	0.04
	Monocyte	0.03	0.03	0.01	0.02
	Myeloid dendritic cell resting	0.01	0.01	0.01	0.01
	NK cell resting	0.00	0.00	0.02	0.03
	Macrophage M2	0.27	0.10	0.21	0.09
CIBERSORT-ABS	Mast cell activated	0.04	0.03	0.00	0.01
	B cell plasma	0.09	0.05	0.02	0.02
	B cell naive	0.05	0.06	0.01	0.01
	T cell CD4+ memory resting	0.13	0.05	0.06	0.04
	Monocyte	0.02	0.03	0.01	0.01
	Myeloid dendritic cell resting	0.01	0.01	0.00	0.00
	Macrophage M2	0.17	0.05	0.08	0.07
	Macrophage M0	0.00	0.00	0.05	0.05
	NK cell activated	0.02	0.02	0.01	0.01
	B cell memory	0.01	0.02	0.00	0.01
	NK cell resting	0.00	0.00	0.01	0.01
EPIC	B cell	0.02	0.03	0.00	0.01
	T cell CD4+	0.05	0.02	0.03	0.01
	T cell CD8+	0.02	0.01	0.01	0.01
	Endothelial cell	0.03	0.01	0.02	0.01

	Macrophage	0.01	0.00	0.01	0.01
MCPCOUNTER	Neutrophil	31.45	16.61	7.03	4.99
	Myeloid dendritic cell	6.28	2.84	1.30	1.16
	B cell	14.77	16.54	2.01	3.33
	Cancer associated fibroblast	743.13	508.12	297.41	317.95
	Endothelial cell	9.56	3.18	6.07	3.39
	T cell	4.99	1.80	3.65	1.74
	cytotoxicity score	3.16	0.96	1.88	1.71
	Monocyte	10.43	3.45	6.78	4.96
	Macrophage/Monocyte	10.43	3.45	6.78	4.96
QUANTISEQ	uncharacterized cell	0.64	0.06	0.82	0.05
	T cell CD4+ (non-regulatory)	0.07	0.03	0.02	0.02
	B cell	0.03	0.03	0.00	0.01
	Neutrophil	0.11	0.04	0.06	0.02
	Macrophage M1	0.08	0.02	0.05	0.02
	Macrophage M2	0.03	0.01	0.02	0.01
	T cell regulatory (Tregs)	0.02	0.01	0.01	0.01
TIMER	T cell CD8+	0.27	0.02	0.17	0.04
	Myeloid dendritic cell	0.65	0.09	0.50	0.09
	B cell	0.17	0.08	0.10	0.04
	Macrophage	0.09	0.04	0.04	0.03
XCELL	B cell memory	0.08	0.08	0.00	0.01
	Class-switched memory B cell	0.05	0.03	0.01	0.01
	Cancer associated fibroblast	0.16	0.11	0.02	0.04
	B cell	0.15	0.17	0.02	0.03
	microenvironment score	0.25	0.13	0.06	0.07
	stroma score	0.09	0.06	0.02	0.03
	immune score	0.16	0.12	0.04	0.05
	B cell naive	0.02	0.04	0.00	0.00
	Hematopoietic stem cell	0.17	0.07	0.07	0.05
	T cell CD4+ Th1	0.01	0.02	0.07	0.04
	B cell plasma	0.02	0.01	0.01	0.01
	Macrophage M1	0.02	0.01	0.01	0.01
	Myeloid dendritic cell activated	0.26	0.06	0.14	0.10
	Plasmacytoid dendritic cell	0.03	0.02	0.01	0.02
	Myeloid dendritic cell	0.02	0.01	0.01	0.01
	T cell CD8+ central memory	0.02	0.02	0.01	0.01
	Granulocyte-monocyte progenitor	0.01	0.02	0.00	0.01
	T cell CD4+ memory	0.04	0.02	0.03	0.02
	Macrophage	0.02	0.01	0.01	0.02
